# Surface Acoustic Waves (SAW)-Based Biosensing for Quantification of Cell Growth in 2D and 3D Cultures

**DOI:** 10.3390/s151229909

**Published:** 2015-12-19

**Authors:** Tao Wang, Ryan Green, Rajesh Ramakrishnan Nair, Mark Howell, Subhra Mohapatra, Rasim Guldiken, Shyam Sundar Mohapatra

**Affiliations:** 1 Center for Research and Education in Nanobioengineering, University of South Florida, Tampa, FL 33612, USA; taowang@mail.usf.edu (T.W.); rgreen1@health.usf.edu (R.G.); rrnair@gmail.com (R.R.N.); mhowell1@health.usf.edu (M.H.); 2 Microfluidics and Acoustics Laboratory, Department of Mechanical Engineering, College of Engineering, University of South Florida, Tampa, FL 33612, USA; 3 Department of Molecular Medicine, University of South Florida, Tampa, FL 33612, USA; 4 Departments of Internal Medicine, University of South Florida, Tampa, FL 33612, USA; 5 Transgenex Nanobiotech Inc, Tampa, FL 33612, USA

**Keywords:** surface acoustic waves (SAW), shear horizontal—surface acoustic waves (SH-SAW), Biosensor, zinc oxide (ZnO), cancer, 3D cell culture

## Abstract

Detection and quantification of cell viability and growth in two-dimensional (2D) and three-dimensional (3D) cell cultures commonly involve harvesting of cells and therefore requires a parallel set-up of several replicates for time-lapse or dose–response studies. Thus, developing a non-invasive and touch-free detection of cell growth in longitudinal studies of 3D tumor spheroid cultures or of stem cell regeneration remains a major unmet need. Since surface acoustic waves (SAWs) permit mass loading-based biosensing and have been touted due to their many advantages including low cost, small size and ease of assembly, we examined the potential of SAW-biosensing to detect and quantify cell growth. Herein, we demonstrate that a shear horizontal-surface acoustic waves (SH-SAW) device comprising two pairs of resonators consisting of interdigital transducers and reflecting fingers can be used to quantify mass loading by the cells in suspension as well as within a 3D cell culture platform. A 3D COMSOL model was built to simulate the mass loading response of increasing concentrations of cells in suspension in the polydimethylsiloxane (PDMS) well in order to predict the characteristics and optimize the design of the SH-SAW biosensor. The simulated relative frequency shift from the two oscillatory circuit systems (one of which functions as control) were found to be concordant to experimental data generated with RAW264.7 macrophage and A549 cancer cells. In addition, results showed that SAW measurements *per se* did not affect viability of cells. Further, SH-SAW biosensing was applied to A549 cells cultured on a 3D electrospun nanofiber scaffold that generate tumor spheroids (tumoroids) and the results showed the device's ability to detect changes in tumor spheroid growth over the course of eight days. Taken together, these results demonstrate the use of SH-SAW device for detection and quantification of cell growth changes over time in 2D suspension cultures and in 3D cell culture models, which may have potential applications in both longitudinal 3D cell cultures in cancer biology and in regenerative medicine.

## 1. Introduction

Detection and quantification of cell viability and growth in two-dimensional (2D) and three-dimensional (3D) cell cultures commonly involve harvesting of cells and therefore requires a parallel set-up of several replicates for time-lapse or dose–response studies. Currently, cell growth or proliferation of flat 2D cultures utilize MTT assay, flow cytometry and Ki67 staining. Similarly, measuring cell growth and proliferation in 3D cultures consist of terminal studies that may include trypsinization and staining with trypan blue and quantification. Thus, a non-invasive and touch-free detection of cell growth or proliferation in longitudinal studies, especially for 3D tumor spheroid cultures and stem cell regeneration remains a major unmet research need.

Surface acoustic waves (SAWs) have been broadly applied in many areas of micro-sensor technology. Gas sensors [1], biosensors [2,3] and chemical sensors [4] are a few of the leading applications for SAW sensors. Generally, biosensors are widely used in cancer biomarker detection and bio-agent detection. Due to SAWs’ advantages of low cost, small size and ease of assembly, SAW-based biosensor technologies have the potential to transform the cancer and bio-agent detection fields [3,5]. However, the potential application of SAWs for the detection of cell growth has not been reported and remains to be elucidated.

SAWs consist of two particle displacement components. One is along the direction of wave propagation and the second one is normal to the surface, such as Rayleigh waves. Rayleigh waves, which generate compressional waves, are affected and damped by the liquid loading and dissipate the wave energy into the liquid. Therefore Rayleigh surface acoustic waves are less sensitive to mass loading changes [6]. Shear horizontal-surface acoustic waves (SH-SAW) with the substrate polarized normal to wave propagation are most commonly used in sensor applications that involve fluidics. Many different wafer types with special cuts are used for shear horizontal wave excitation, such as ST-cut Quartz [2,7,8] and 36°Y-cut LiTaO_3_ [6,9,10]. ST-cut Quartz and 36°Y-cut LiTaO_3_ are very stable substrates for sensor applications. However, the electroacoustic coupling coefficient (*K*^2^) of ST-cut Quartz is much smaller than that of 36°Y-cut LiTaO_3_ (36°Y-cut LiTaO_3_ is 4.7 [11] and ST-cut Quartz is 0.0016 [12]).

Because of its high electroacoustic coupling coefficient, the 36°Y-cut LiTaO_3_ generates more stable signals when the SH-SAWs travel through polydimethylsiloxane (PDMS), which absorbs the majority of the energy generated by the interdigital transducers [13,14]. PDMS has been widely used in biomedical devices due to its biocompatibility and ease of manufacture into fluidic channels. An optimization of the PDMS channel sidewall thickness was demonstrated to reduce the damping effect of the PDMS on the wave propagation [15], thereby increasing the sensitivity of the sensor. Even though 36°Y-cut LiTaO_3_ has a higher electroacoustic coupling coefficient, it also has a higher temperature coefficient compared to the ST-Quartz. Various guide layers can be deposited on the LiTaO_3_ to change the phase velocity and temperature coefficient of the system. Zinc Oxide (ZnO) is a relatively common material in sensor and SAW fields. The majority of SAW devices coated with ZnO are used as Ph [16] or UV sensors [17]. Coating a ZnO layer on a LiTaO_3_ substrate reduces the temperature coefficient and increases the mass sensitivity [18,19,20,21], hence addressing the shortcoming of the LiTaO_3_ substrate as opposed to its alternatives.

We have been investigating the possibility of non-invasive touch-free monitoring of cell proliferation/growth in long-term 2D and 3D cell cultures. Serendipitously, we discovered that SAW-based biosensing produced a different frequency shift. We reasoned that SH-SAW using 36°Y-cut LiTaO_3_ wafers coated with ZnO might have the potential to measure and quantify cellular mass changes. To test this idea, we utilized a PDMS channel/well and surface acoustic wave transducers coated with a ZnO layer to measure mass changes due to increasing cell numbers in normal murine RAW264.7 macrophages and human A549 lung adenocarcinoma cell lines. Our results indicate that the proposed microfluidic SAW device is capable of monitoring and quantifying cell density of both cell lines in suspension as well as cultured on a 3D-nanofiber scaffold.

## 2. Working Principle

The frequency of the SH-SAW shifts when the waves travel through the media inside the PDMS well due to propagation loss. When the substrate where the surface acoustic wave propagates is liquid loaded, the phase velocity and attenuation of the wave can be related to the mechanical properties of the media such as viscosity and density, and electrical characteristics such as permittivity and conductivity. The perturbation formula was originally derived from Auld’s perturbation theory which applies to gas sensors and then extended to liquid phase applications by J Kondoh *et al*. [4,22,23].

Change in velocity:

(1) $$\frac{\Delta V}{V}=-\frac{K_{S}^{2}}{2}\frac{{\left(\sigma_{1}/\omega \right)}^{2}+\left(\varepsilon_{1}-\varepsilon_{REF}\right)\left(\varepsilon_{1}+\varepsilon_{PIEZO}\right)}{{\left(\sigma_{1}/\omega \right)}^{2}+{\left(\varepsilon_{1}+\varepsilon_{PIEZO}\right)}^{2}}$$

Change in attenuation:

(2) $$\frac{\Delta \alpha }{k}=\frac{K_{S}^{2}}{2}\frac{\left(\sigma_{1}/\omega \right)\left(\varepsilon_{REF}+\varepsilon_{PIEZO}\right)}{{\left(\sigma_{1}/\omega \right)}^{2}+{\left(\varepsilon_{1}+\varepsilon_{PIEZO}\right)}^{2}}$$

In the equations, $K_{S}^{2}$ represents the electromechanical coupling factor of the substrate, $\varepsilon_{PIEZO}$ is the effective dielectric constant, $\varepsilon_{1}$ is the dielectric constant of sample liquid, $\varepsilon_{REF}$ is the dielectric constant of the reference liquid, $\sigma_{1}$ is the conductivity of the sample liquid, and ω is the angular frequency of the SH-SAW. Based on the equation above, mixtures containing particles of different density can easily be distinguished by the SH-SAW device.

## 3. Design and Fabrication of Bio-Sensor

### 3.1. Device Design

**Figure 1:**
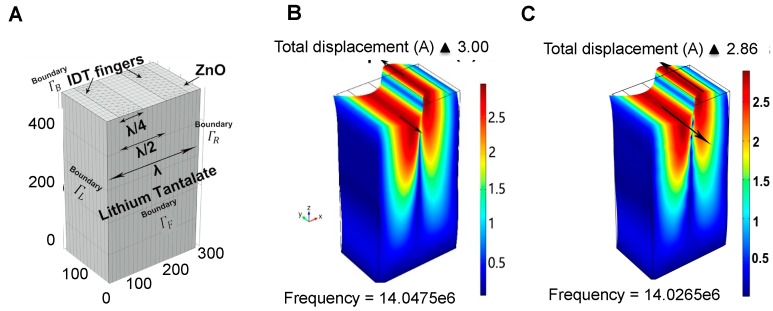
A 3D COMSOL model and simulation results based on the 36°Y-cut LiTaO3: (**A**) 3D cell model geometry with mesh; (**B**) resonance frequency of the IDTs with a 200 nm thick ZnO layer; and (**C**) resonance frequency of the IDTs with 12.5 K cells media on the 200 nm thick ZnO layer surface.

A 3D COMSOL model that consists of a two-port resonator was built to characterize changes to the wave propagation characteristics resulting from alterations to mechanical properties inside the well. A simplified 3D cell model of the Lithium tantalate resonator was built to obtain the resonator frequency shifts by the Eigen frequency module of the COMSOL software. The individual 3D cell was set as periodic to simulate the entire SAW sensor with a fairly-simplified geometry. Figure 1 illustrates one wavelength cell of the simulated design with interdigital transducers (IDTs). Two interdigital transducer fingers are illustrated where one of them was connected to the ground.

After the model was built and material properties were applied, a mesh was created with total degrees of freedom of 679,924. The mesh consisted of 111,679 domain elements, 28,942 boundary elements and 2892 edge elements. 36°Y-cut LiTaO_3_ with or without a ZnO coating was employed as the choice of substrate to simulate the resonator frequency. The simulation results indicated that the operation frequency would be 14.0475 MHz without ZnO while the fabricated operation frequency was experimentally measured as 14.056 MHz. The simulation result of the 3D-cell with ZnO is 14.03296 MHz, while the experimentally measured value is 14.04120 MHz, illustrating the validity of the developed simulation designs. As expected, the shear horizontal wave propagated in the *x* direction with the substrate polarized in the *y* direction, as illustrated in Figure 1B,C. Additional device design details are given in Table 1.

**Table 1:** Device Parameters used for the simulation and fabrication of the IDT transducers.

PARAMETERS	SETTINGS
Wavelength (λ)	297 μm
Number of reflecting fingers	30 pairs
Finger width	74.25 μm
Wavelength of reflecting fingers	297 μm
Number of fingers	30 pairs
Well diameter	6.5 mm
SAW velocity	4160 m/s
ZnO layer thickness	200 nm
Finger height	100 nm
Operation frequency	14.05 MHz

### 3.2. Device Fabrication

The IDTs were fabricated by the traditional micro-lithography methods while the microfluidic well was fabricated by the conventional PDMS micro molding technique. Further details on the fabrication process can be found in our recent reports [24,25,26]. After the IDTs were fabricated on the lithium tantalate substrate, ZnO sputtering was carried out. A 200 nm thick ZnO film was deposited at 150 °C in 2.5 h. After the ZnO deposition, the PDMS well was bonded to the lithium tantalate substrate after being exposed to 30 s oxygen plasma for increased bonding.

**Figure 2:**
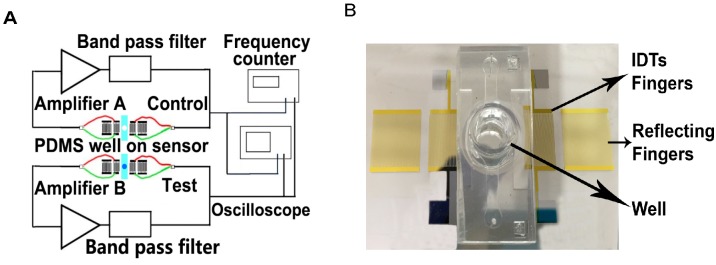
(**A**) Conceptual view of the oscillatory circuit system. Two resonators with custom-designed oscillatory circuit system were used with one of them as control group; (**B**) Fabricated and assembled resonator and fluidic well.

The SAW resonator can be used as a propagation delay-line with a pair of IDT transducers that serve to excite and receive the acoustic wave. Therefore a custom-designed oscillatory circuit system was used for quantifying the cell concentrations as shown in the conceptual view in Figure 2. Compared to other detection methods such as network analyzer, an oscillatory circuit was employed as it offers higher stability as well as higher sensitivity [3,27]. In the oscillator circuit detection system, the SAW sensor was employed as the feedback element of the RF amplifier. The relative change of SAW velocity due to mechanical and electrical changes resulted in an oscillation frequency shift. These changes in oscillation frequency were detected with a digital frequency counter. The setup used two variable gain RF amplifiers (Olympus 5073PR and Olympus 5072PR, Olympus NDT Inc., Waltham, MA, USA), a digital frequency counter (Agilent 53220A, Agilent Technologies Inc, Santa Clara, CA, USA), an oscillator (Tektronix TDS2001C, Tektronix Inc., Beaverton, OR, USA), as reported previously [3]. A band pass filter was used on the amplifier to eliminate the frequencies lower than 5 MHz and higher than 20 MHz in the loop. The two oscillation loops were employed to minimize the background noise and relate the frequency shift to the mass loading of different cell concentrations. A constant volume of different cell concentrations media was supplied to the well in the test loop for each experiment. During the experiments, the frequency changes for the test group while the frequency of the control group remained nearly constant. From the perturbation theory, when the surface acoustic waves propagates thru the detection area of the sensor, the phase velocity changes due to mass loading from the cell media. In Equations (3) and (4) presented below, $V_{1}$ is the surface wave phase velocity of the control group device and $V_{2}$ is the surface wave phase velocity of the actual tested device.$V_{2}^{'}$ represents the phase velocity of the surface acoustic wave travelling through different cell concentrations. During the experiments, the only real time relative frequency $\frac{f_{2}}{f_{1}}$ was recorded by the frequency counter. Then the data were sorted by Matlab^©^ and plotted out in normalized relative frequency shift.

(3) $$\frac{\Delta V}{V}=\frac{V_{2}-V_{2}^{'}}{V_{1}}$$

(4) $$\frac{\Delta f}{f}=\frac{\Delta V}{V}=\frac{f_{2}-f_{2}^{'}}{f_{1}}$$

## 4. Experimental Setup 

### 4.1. Experimental Protocol for SAW Measurement

A549 human lung adenocarcinoma cells were maintained in RPMI media containing 10% fetal bovine serum (FBS) and 1% penicillin streptomycin. RAW-264.7 murine macrophages (used as an example of a non-cancerous cell) were maintained in Dulbecco’s modified eagle medium (DMEM) media containing 10% FBS and 1% penicillin streptomycin. All cells were cultured in a humidified incubator at 37 °C in a 5% CO2 atmosphere. Cells were collected via trypsinization and counted using a hemocytometer. For SAW measurement of cells in suspension, cell suspensions of decreasing concentration were prepared by serial dilution in phosphate buffered saline (PBS) containing 1% FBS. Half a minute after the frequency counter started to record, 100 µL of each suspension was placed on the chip of test group to record the relative frequency response for a duration of 10 min. After recording each sample, the cell suspension was removed by vacuum and the well was washed with three changes of PBS followed by three changes of water to clean the sensing area.

### 4.2. Experimental Protocol for Measuring Cell Viability

We determined the cell viability using trypan blue staining in combination with a T20^TM^ automated cell counter (Bio-Rad). Cell suspension was mixed with trypan blue at a ratio of 1:1 respectively and the resulting cell suspension was loaded on to a cassette for measurement in the cell counter. The T20^TM^ uses microscopy in conjunction with an algorithm to calculate the total cell count and assesses the cell viability by trypan blue exclusion without any interference from the user. The advantage of this methodology is that it ensures reproducibility in the cell count independent of the users. Additionally, we validated the instrument for measuring the cell viability by using a cell suspension sample that was heated for 15 min at 56 °C to induce cell death and show that the cell counter was able to detect increase in cell death (Supplementary Material Figure 1).

### 4.3. Experimental Protocol for Measuring Cell Proliferation

To examine any long term effects on cell proliferation a re-plating experiment was performed in which cells were collected and then seeded onto a 96 well culture plate after SAW measurements and allowed to grow for three days. Cell number and morphology were compared to untested control cells by both light microscopy (Olympus BX51) and by staining the cells with Hoechst 33342 (NucBlue, Life Technologies) and then capturing images using fluorescence microscopy (Olympus BX51). This assay was designed to reveal any changes to the cell proliferation rate as a result of SAW measurement.

### 4.4. Experimental Protocol for Culturing 3D Tumoroids

For growing cell cultures in three-dimensions (3D), we used a fibrous scaffold developed by our lab (3P scaffold) which promotes the growth of 3D “tumoroids” when seeded with cancer cells. 3P scaffold was prepared by electrospinning as described previously [28]. Scaffold was placed into a 96 well plate and 5000 A549 cells were seeded into each well in RPMI media. Cells were allowed to grow on the scaffold for eight days and the culture media was changed every two days. Successful growth of cells in 3D was confirmed by staining the cells with Hoechst 33342 (NucBlue, Life Technologies) and then capturing images using fluorescence microscopy (Olympus BX51).

## 5. Results and Discussion

### 5.1. Cell Viability and Cell Proliferation Is Not Affected after SAW Measurements

**Figure 3:**
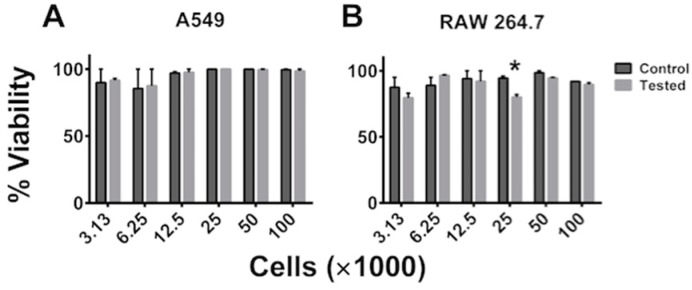
Cell viability following SAW measurement. Immediately following SAW measurement the viability of (**A**) A549 and (**B**) RAW 264.7 cells was determined by trypan blue exclusion. Data is plotted as mean ± standard error of the mean. The data is a representative of a study that was performed in triplicates and performed at least two independent times. A student’s *t*-test was used to evaluate significance (* *p* ≤ 0.05, when compared to control).

One of our first concerns was that the cellular stress due to seeding of the cells in our device followed by exposure to acoustic waves would have a negative impact on the cells’ viability and their ability to proliferate. To demonstrate the procedure’s innocuous nature and thus its utility in translational lab setting, we determined cell viability as described in the experimental setup. We tested the viability of both normal (RAW 264.7) and cancerous (A549) cells immediately following SAW measurement by trypan blue exclusion. Three replicates were tested for each concentration of both cell lines. A student’s *t*-test was used to determine any significant difference in viability between each pair of control and SAW tested groups. A *p*-value ≤ 0.05 was considered to be significant. As seen in Figure 3, out of the 12 groups compared, only one showed any significant difference in viability.

**Figure 4:**
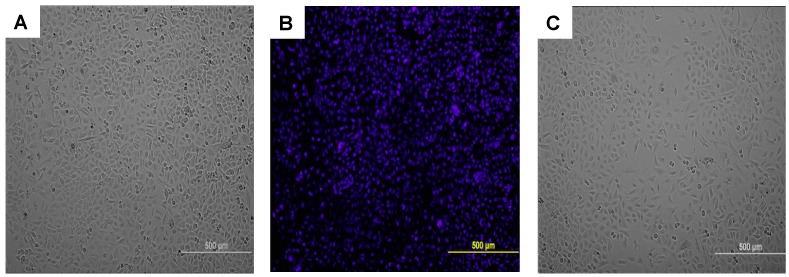
Cell proliferation following SAW measurement. Immediately following SAW measurement A549 cells were seeded onto a 96-well plate at a density of 5000 cells per well. Representative images of control and tested groups are shown: (**A**) A549 cells 72 h after SAW test; (**B**) NucBlue staining of A549 cells 72 h after test; and (**C**) Control cells after 72 h.

Next, we looked at the long-term effect of the SAW measurement on cell proliferation by performing a re-plating assay as described earlier. Briefly, A549 cells were collected and then seeded onto a 96-well culture plate after SAW measurements. As seen in Figure 4, A549 cells exposed to SAW (Figure 4A) and control untested cells which were not exposed to the SAW device (Figure 4C) reveals no obvious changes to cell morphology or growth rate after 72 h. The nuclear staining in Figure 4B shows that cells have in-tact nuclei and appear healthy.

### 5.2. Frequency Shift Increases with Increasing Cell Concentration and Sensitivity Is Further Aided by the Use of ZnO

**Figure 5:**
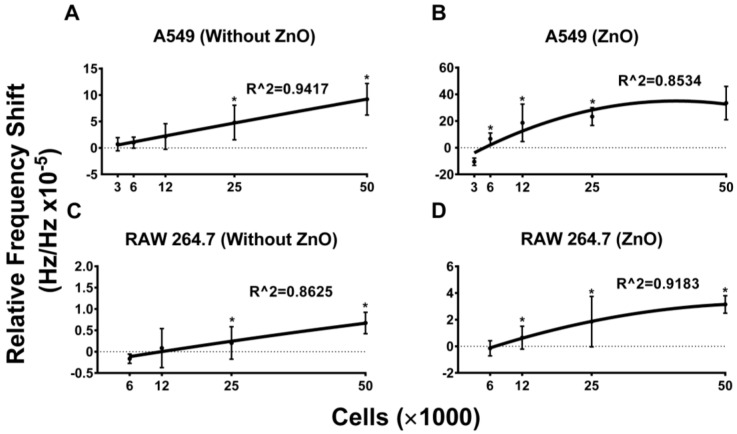
Relative frequency shift response to the different cell concentrations. (**A**,**C**) The bare SAW resonator response to cell with concentrations of 3000, 6250, 12,500, 25,000 and 50,000 in 100 µL media for each test, A549 (**A**) and RAW 264.7 (**C**). (**B**,**D**) The SAW resonator coating with ZnO layer response to cells with concentration of 3000, 6250, 12,500, 25,000 and 50,000 in 100 µL media for each test, A549 (**B**) and RAW 264.7 (**D**). Data are plotted as mean ± 95% CI. The data are representative of a study that was performed in triplicates and performed at least two independent times. A student’s *t*-test was used to evaluate significance (*p* ≤ 0.05). * indicates a significant difference in the frequency shift between the labeled group and the adjacent lower concentration. Best fit curves were calculated using a second order polynomial model in the GraphPad Prism software application.

Once we confirmed that our bio-sensing device and measurement protocol was bio-compatible, we next wanted to determine the sensitivity of our device in accurately measuring cell concentrations. For this we used two variations of our device, one that was coated with ZnO and the second that was kept bare. The SAW measurement protocol for both the devices was kept constant as described in the experimental setup section. Both the non-cancerous (RAW 264.7) and cancerous (A549) cells were examined in the devices at 6250, 12,500, 25,000 and 50,000 cells per 100 μL. The concentration of cells were chosen based on cell density that we would encounter when performing actual research studies. As seen in Figure 5, we saw a cell dependent increase in the frequency shift in both the cell lines tested. Interestingly, the layer of ZnO increased the sensitivity of the device in recording changes in cell numbers in both of the cell lines tested. Specifically, comparing sensors containing the ZnO layer (Figure 5B,D) to those with the bare substrate (Figure 5A,C) revealed that the ZnO layer increased the relative frequency response by four times which means it increased the sensitivity of the device.

### 5.3. SAW Measurements of Cell Density Match Simulation Results

Having confirmed that our device accurately distinguishes between cell numbers, we further optimized our device for future experimental protocols. For this, we decided to establish a working theoretical model based on the 3D COMSOL model by adding mass loading module that best mimicked our bio-sensing device. One advantage of having such a model is that it will enable us to tweak several parameters on our device and run a simulation experiment without having to spend time and money on actual experiments. Towards this goal, we set up the simulation studies, wherein the weight of each individual cell was assumed to be 1 pg and different concentrations of cells were added to the ZnO surface (thickness 200 nm) on 36°Y-cut LiTaO_3_ substrate by modifying the mass loading in the developed model. The cell concentrations simulated were 0, 6250, 12,500, 25,000, 50,000, and 100,000 cells/microwell. After the cell (mass) loading was applied, the relative frequency response to the different cell concentrations was simulated (Figure 2).

**Figure 6:**
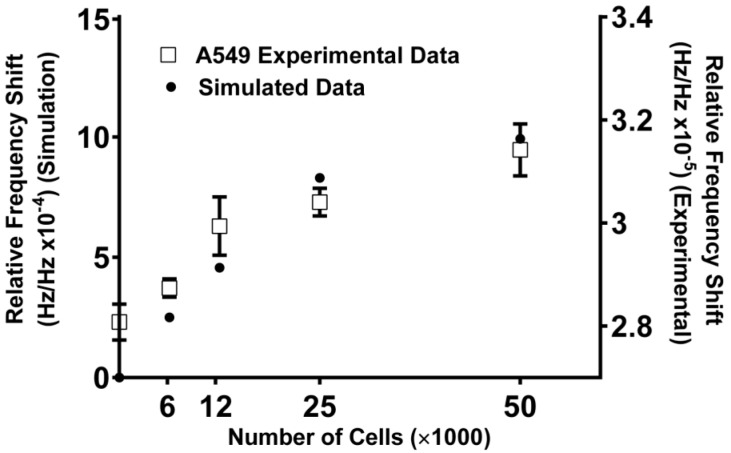
SAWs experiment data match the tendency of the simulation results on the ZnO coated sensor. Experimental data are plotted as mean ± standard error of the mean The data is a representative of a study that was performed in triplicates.

In order to normalize the frequency shift obtained in response to the cell concentration chance, relative frequency response, which is the frequency shift over the operation frequency (Δf/f), was plotted. With the cell concentration increasing (hence the mass), the phase velocity of the substrate decreased which resulted in decreasing frequency. The simulated relative frequency shift was found to be about one order of magnitude higher than the experimental results, without applying any to the simulation when presenting the results. The mismatch between the raw simulation data presented and the experimental data is expected which may be attributed to the following factors. First, the weight of the individual cell was assumed to be 1pg in the simulations for referencing purposes, whereas in actual experimentation the weight of the cells can differ significantly depending on the cell type. Second, the cells with media were placed on the bottom of the well in the actual experiments performed. On the other hand the mass loading on the entire sensor chip surface was simulated for the 3D COMSOL model. Nonetheless, there is a very good concordance of the simulated and experimental data, with a very similar match in the trends obtained (Figure 6). One can easily apply a correction factor to the simulation for the specific cell lines or application if closer frequency shift magnitude is needed.

### 5.4. SAW Measurements Aid in Monitoring Growth of A549 3D Spheroid Cultures

**Figure 7:**
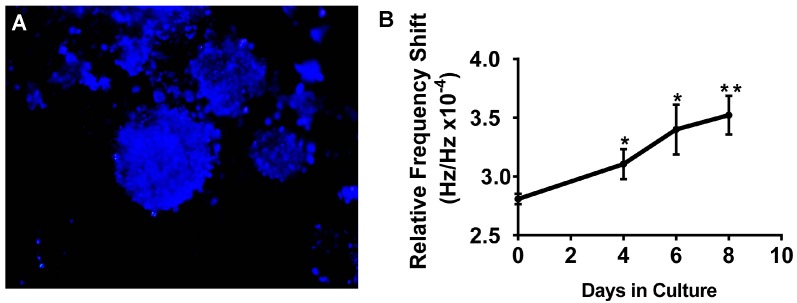
(**A**) Representative image of A549 cells growing in 3D tumoroid structures on the 3P scaffold using NucBlue nuclear stain on Day 8 of culture. (**B**) A549 cells were cultured on the 3P scaffold for eight days. On Days 0 (scaffold with no cells), 4, 6, and 8 scaffolds were collected and transferred to the SAW device with ZnO layer for measurement. Data is plotted as mean ± standard error of the mean. The data is a representative of a study that was performed in triplicates. * = significant increase from Day 0; ** = significant increase from Day 4 (*p* ≤ 0.05).

Use of 3D cell culture techniques in cancer research is rapidly expanding due to the limited ability of traditional 2D culture to accurately model *in vivo* cell behavior. To test whether the SAW device is able to measure the cell density of 3D spheroids, (also referred to as tumoroids) growing on a fiber matrix we cultured A549 cells on the matrix. A549 cells were allowed to grow on the scaffold for eight days and the culture media was changed every two days (Figure 7A). The 3P scaffold alone was first measured as control and corresponding reading was designated as Day 0. On Days 4, 6, and 8 scaffolds were removed from the plate and assayed on the SAW sensor. Data shown in Figure 7B demonstrate that the sensor was able to detect the change in density resulting from cell proliferation over time in the 3D environment. There was a linear increase in frequency shifts observed in A549 tumoroids with time. This increase was similar to increases in tumoroid size and number reported previously for other cancer cell lines [28].

## 6. Conclusions

Based on these results, the acoustic measurement procedure seems to have no ill effect on cell viability in either the A549 cancer cell line or the RAW 264.7 macrophages. Cell proliferation was also unaffected by SAW measurements in A549. The device’s ability to detect changes in cell density on the 3D scaffold over time along with its biocompatibility reveal great potential for this device to be incorporated into 3D *in vitro* cancer models. The platform thus created would enable continuous real-time measurement of cell growth in a 3D environment during bio assays including drug screens, multi cell co-cultures, gene knockdown/knockout, *etc.*

There are several potential translational implications for these. Acoustic biosensing involves a highly sensitive and tunable SAW (37–46), which can be performed without any electrode touching the tumoroids and acoustical response can be acquired independent of existence of a magnetic/electrical field and iron oxide/MnO nanoparticles in the flow field. The potential for miniaturization and integration of complex functions into “multi-cell tumoroids on chip” exists, which is expected to revolutionize real-time tracking of biomarkers and clinical diagnostics and prognostics of cancers in a point-of-care setting for personalizing therapy. In addition, monitoring of physiologic/metabolic tumor markers via acoustic bio-sensing is expected to increase resemblance of tumoroid cultures to *in vivo* tumors and provide a precise, stable, and well-defined culture environment for cellular assays.

## Supplementary Files

Supplementary File 1
