# Supplementary material for: Surface Acoustic Waves (SAW)-Based Biosensing for Quantification of Cell Growth in 2D and 3D Cultures
Source: Sensors (Basel). 2015 Dec 19;15(12):32045–55. doi: 10.3390/s151229909 (PMC4721826; doi:10.3390/s151229909)
Supplement: Supplementary File 1 [file sensors-15-29909-s001.pdf]

Supplementary Materials: Surface Acoustic Waves (SAW)-Based Biosensing for Quantification of Cell Growth in 2D and 3D Cultures

Tao Wang [1,2,†], Ryan Green [1,3,†], Rajesh Ramakrishnan Nair [1,4,5,†], Mark Howell [1,3], Subhra Mohapatra [1,3,*], Rasim Guldiken [1,2,*] and Shyam Sundar Mohapatra [1,4,*]

Quantification of Cell Growth in 2D and 3D Cultures

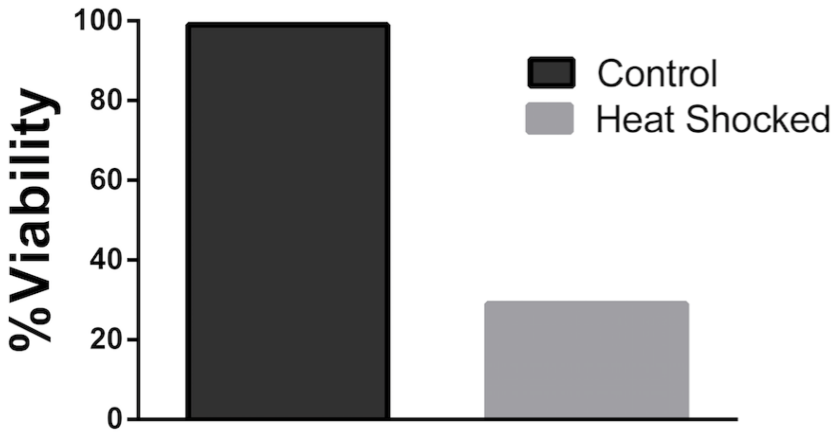

Figure S1. A549 cells where either left untreated (Control) or subjected to heat (Heat Shocked) at 56 °C for 15 min. At the end of the heat shock the cells were mixed with trypan blue solution and processed for cell counting using the T20™ automated cell counter according to the manufacturer's instructions (Bio-Rad). The data was plotted as % viability wherein the viability of the control cells was set at 100%.
